# Performance Analysis of Cooperative NOMA Networks with Imperfect CSI over Nakagami-*m* Fading Channels

**DOI:** 10.3390/s20020424

**Published:** 2020-01-11

**Authors:** Xianli Gong, Xinwei Yue, Feng Liu

**Affiliations:** 1 School of Electronic and Information Engineering, Beihang University, Beijing 100191, China; gongxl@buaa.edu.cn; 2 School of Information and Communication Engineering, Beijing Information Science and Technology University, Beijing 100101, China

**Keywords:** cooperative non-orthogonal multiple access, decode-and-forward, imperfect channel state information, outage probability

## Abstract

In this paper, we investigate a downlink cooperative non-orthogonal multiple access (NOMA) network with decode-and-forward relaying, where two scenarios of user relaying with direct link and user relaying without direct link are discussed in detail. More particularly, the performance of cooperative NOMA system under the assumption of imperfect channel state information (ipCSI) is studied over Nakagami-*m* fading channels. To evaluate the outage performance of the above discussed two scenarios, the closed-form expressions of outage probability for a pair of users are derived carefully. The diversity orders of users are achieved in the high signal-to-noise region. An error floor appears in the outage probability owing to the existence of channel estimation errors under ipCSI conditions. Simulation results verify the validity of our analysis and show that: (1) NOMA is superior to conventional orthogonal multiple access; (2) The best user relaying location for cooperative NOMA networks should be near to the base station; and (3) The outage performance of distant user with direct link significantly outperforms distant user without direct link by comparing the two scenarios.

## 1. Introduction

In order to improve spectral efficiency and meet the needs of users for massive connectivity, non-orthogonal multiple access (NOMA) has attracted great attention from many researchers [1,2]. NOMA is widely regarded as a promising multiple access technique for the fifth-generation mobile communication networks [3]. The key idea of NOMA is that multiple users can be served by sharing the same physical resource over different power levels [4]. More specifically, multiple users’ signals are transmitted by employing the superposition coding scheme at the transmitter and these users’ signals are decoded by applying successive interference cancellation at the receiver [5]. To ensure user fairness, more transmit power is allocated to users with worse channel conditions, where NOMA is capable of providing services for multiple users.

Cooperative NOMA is a promising technology in the future wireless network, which has improved the spectral efficiency and enhanced the reliability of wireless network. The current research on cooperative NOMA is divided into two aspects. One aspect is that the nearby NOMA user with better channel conditions is viewed as user relaying to forward the information to the distant NOMA users [6,7,8,9,10]. In Reference [6], the authors initially have proposed cooperative NOMA scheme in which the nearby users with better channel conditions are regard as decode-and-forward (DF) relaying to improve the system reliability. From the perspective of energy efficiency, cooperative NOMA with simultaneous wireless information and power transfer (SWIPT) has been studied in Reference [7], in which the nearby NOMA user plays the role of an energy harvesting DF relaying to assist distant NOMA user. The performance of integrating cooperative NOMA with full-duplex (FD) device-to-device (D2D) communication has been researched in Reference [8], in which the NOMA-strong user is employed to assist the NOMA-weak user by FD D2D communications. Considering a two-user NOMA network, the best-near best-far user selection scheme has been developed to study the outage performance of NOMA-based cooperative relaying systems in Reference [9], where the best cell-center user is selected to act as an energy harvesting relay to help a selected cell-edge user. The performance of cell-edge users in multiple-input single-output NOMA systems has been researched in Reference [10] by using transmit antenna selection and SWIPT-based cooperative transmission, in which the cell-center user is considered as a hybrid time-switching/power-splitting energy harvesting relay. The other aspect is that the relay is introduced in the NOMA system, where the relay is an amplify-and-forward (AF) relay, DF relay, or an opportunistic relay [11,12,13]. The outage performance of cooperative NOMA networks with SWIPT has been investigated in Reference [11], where the DF relay is introduced to serve as an energy harvesting relay to deliver information to users. The two-stage DF and AF relay selection schemes for cooperative NOMA have been proposed in Reference [12], and two optimal relay selection schemes for downlink cooperative NOMA system have been proposed in Reference [13], in which one relay is chosen from multiple relays to communicate with the users.

The existing works on cooperative NOMA are analyzed under two conditions. The first condition is perfect channel state information (pCSI) [14,15]. Outage probability of a fixed gain NOMA based AF relaying system has been investigated under pCSI conditions over Nakagami-*m* fading channels in Reference [14]. The performance of a NOMA-based cooperative relaying system has been investigated under pCSI conditions over Rician fading channels in Reference [15], and the exact expression of average achievable rate has been derived. The second condition is imperfect channel state information (ipCSI) [16,17]. The authors of Reference [16] considered the ipCSI for a downlink relaying aided NOMA network, where the outage probability of the users has been evaluated in detail. The SWIPT in a multiple-input multiple-output AF relaying system has been investigated under ipCSI conditions in Reference [17], where the relay is an energy harvesting relay and harvests the signal energy transmitted from the source.

The Nakagami-*m* fading channel includes multiple types of channels, and both the Gaussian channel and the Rayleigh fading channel are its special cases. The authors of Reference [18] have studied the performance of a NOMA based AF relaying network, in which NOMA is shown to outperform orthogonal multiple access (OMA) in terms of outage probability and ergodic sum rate and provides better spectral efficiency and user fairness over Nakagami-*m* fading channels. Two NOMA transmission schemes based on different types of relaying in a cooperative NOMA system have been proposed in Reference [19], in which the NOMA-DF scheme can achieve better performance than the NOMA-AF scheme in terms of outage probability and ergodic sum rate over Nakagami-*m* fading channels. The authors in Reference [20] have proposed a NOMA-based transmission scheme in cooperative spectrum-sharing networks, where the NOMA-based scheme is superior to the OMA-based scheme in terms of outage probability and system throughput and provides better spectrum utilization over Nakagami-*m* fading channels. The outage performance of NOMA with fixed power allocation in a downlink NOMA system has been investigated in Reference [21], where NOMA can provide higher individual rates than OMA for the users with larger channel gain over Nakagami-*m* fading channels.

Most of the existing works about cooperative NOMA have been investigated under pCSI conditions over Rayleigh fading channels, but it is difficult to realize in practical wireless systems due to the existence of channel estimation errors. In addition, Nakagami-*m* fading channel is used in many types of fading environments and it have better empirical data comparing with Rayleigh fading channel. The outage performance of cooperative NOMA with user relaying under Rayleigh fading has been studied in Reference [22], but the influence of channel estimation errors on system performance over Nakagami-*m* fading channels has not been taken into account. Motivated by these reasons, we develop this research work.

In this paper, we consider a downlink cooperative NOMA network with ipCSI over Nakagami-*m* fading channels. Two cooperative NOMA transmission scenarios are discussed: (1) The first scenario is that the base station (BS) sends information to distant user through with the aid of nearby user, which is taken as DF relaying; (2) The second scenario is that the BS can not only send information to distant user through nearby user, but also send information directly to distant user. The primary contributions of this paper are summarized as follows:

- We propose a downlink cooperative NOMA network with ipCSI to investigate the effect of the channel estimation errors on system performance in practical wireless communication systems. We analyze the outage performance of NOMA users in two representative cooperative NOMA scenarios in terms of outage probability and diversity order over Nakagami-*m* fading channels.
- We derive the closed-form expressions of outage probability for a pair of NOMA users in the two scenarios of both user relaying without direct link and user relaying with direct link. To better understand the outage behavior of the network, we derive the approximate expressions of outage probability for the pair of NOMA users at high SNR, where we attain the diversity orders of users.
- The simulation results confirm the accuracy of our analysis results and the superiority of NOMA over OMA. We observe that there is the error floor for outage probability at high SNR as a result of channel estimation errors under ipCSI conditions. The outage behaviors of distant user with direct link outperforms distant user without direct link. Additionally, we further observe that the best user relaying location for cooperative NOMA networks should be close to the BS.

To understand NOMA networks investigated in this paper, we compare NOMA with conventional OMA in terms of the aim, solution, pros and cons in Table 1.

The rest of the paper is organized as follows. Section 2 describes the system model. In Section 3, the exact and approximate expressions of outage probability for a pair of NOMA users are derived in two scenarios, and the corresponding diversity orders are analyzed. Numerical results are presented in Section 4 for verifying the accuracy of our analysis. Section 5 concludes the paper.

For the sake of clarity, the main notations and their descriptions are summarized in Table 2.

## 2. System Model

**Figure 1:**
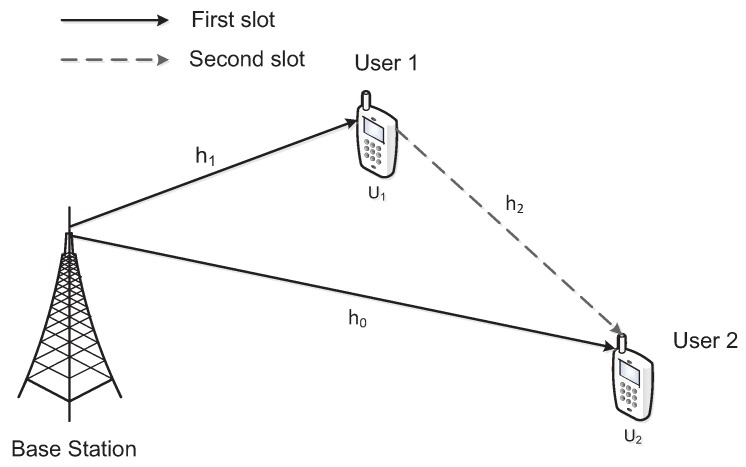
Downlink cooperative NOMA networks.

Consider a downlink cooperative NOMA network, which includes the BS, nearby user $U_{1}$, and distant user $U_{2}$ in a cell, as shown in Figure 1. The BS communicates with $U_{2}$ by utilizing $U_{1}$ as DF relay. The BS, $U_{1}$, and $U_{2}$ are single-antenna devices and operate in half-duplex mode. Assuming that all wireless links suffer from Nakagami-*m* fading and additive white Gaussian noise (AWGN) with zero mean and variance $N_{0}$. Because there are channel estimation errors in wireless networks with ipCSI, the channel coefficient is denoted by $h_{k}$ with fading parameter $m_{k}$ and link average power $E\left({\left|h_{k}\right|}^{2}\right)=\Omega_{k}$ and is modeled as $h_{k}={\hat{h}}_{k}+e_{k}$, k∈{0,1,2}, where ${\hat{h}}_{k}$ denotes the estimated channel coefficient and $e_{k}∼\mathrm{CN}\left(0,\sigma_{e_{k}}^{2}\right)$ represents the channel estimation error which is subject to Gaussian distribution. $h_{0}$, $h_{1}$, and $h_{2}$ denote the channel coefficient of $BS\to U_{2}$, $BS\to U_{1}$, and $U_{1}\to U_{2}$ links, respectively, and ${\hat{h}}_{0}$, ${\hat{h}}_{1}$, and ${\hat{h}}_{2}$ denote the corresponding estimated channel coefficients. $d_{k}$ is assumed to be the distance between two nodes, and we have $\Omega_{k}=d_{k}^{-\alpha }$, where α represents the path loss exponent. When ${\hat{h}}_{k}$ and $e_{k}$ are statistically independent, we have estimated link average power ${\hat{\Omega }}_{k}=\Omega_{k}-\sigma_{e_{k}}^{2}$. Assuming that ηk=σek2σek2ΩkΩk represents the relative channel estimation error, we can obtain $\sigma_{e_{k}}^{2}=\eta_{k}d_{k}^{-\alpha }$ and ${\hat{\Omega }}_{k}=\left(1-\eta_{k}\right)d_{k}^{-\alpha }$.

The BS communicates with distant user $U_{2}$ through direct link $BS\to U_{2}$ and relaying link $U_{1}\to U_{2}$ in a cell. DF protocol is used for the relaying link where nearby user $U_{1}$ acts as user relaying. Two consecutive slots are involved in the whole communication process. In the first slot, the BS transmits superposed signal $\sqrt{a_{1}P_{s}}x_{1}+\sqrt{a_{2}P_{s}}x_{2}$ to relaying user $U_{1}$ and distant user $U_{2}$ according to the principle of NOMA, where $P_{s}$ is the normalized transmission power at the BS, $x_{1}$ and $x_{2}$ are the normalized unit power signals of $U_{1}$ and $U_{2}$, respectively, and $a_{1}$ and $a_{2}$ are the corresponding power allocation coefficients. Assuming that $a_{2}>a_{1}$ and $a_{1}+a_{2}=1$. The received signals at $U_{1}$ and $U_{2}$ are given by

(1) $$\begin{array}{c} y_{U_{1}}=\left({\hat{h}}_{1}+e_{1}\right)\left(\sqrt{a_{1}P_{s}}x_{1}+\sqrt{a_{2}P_{s}}x_{2}\right)+n_{U_{1}} \end{array}$$

and

(2) $$\begin{array}{c} y_{1,U_{2}}=\left({\hat{h}}_{0}+e_{0}\right)\left(\sqrt{a_{1}P_{s}}x_{1}+\sqrt{a_{2}P_{s}}x_{2}\right)+n_{U_{2}} \end{array}$$

respectively, where $n_{U_{1}}$ and $n_{U_{2}}$ are AWGN at $U_{1}$ and $U_{2},$ respectively.

According to NOMA scheme, the nearby user with better channel conditions is allocated less transmit power to achieve fairness between users. Based on the assumption of power allocation coefficients, the signal of $U_{2}$ is decoded firstly by exploiting successive interference cancellation from the received superposed signal at $U_{1}$, where $U_{2}$ with more transmit power has less the inter-user interference. The received signal to interference and noise ratio (SINR) for $U_{1}$ to decode signal $x_{2}$ of $U_{2}$ can be expressed as

(3) $$\begin{array}{c} \gamma_{U_{2}\to U_{1}}=\frac{a_{2}\rho {\left|{\hat{h}}_{1}\right|}^{2}}{a_{1}\rho {\left|{\hat{h}}_{1}\right|}^{2}+\eta_{1}d_{1}^{-\alpha }\rho +1}, \end{array}$$

where $\rho =\frac{P_{s}}{N_{0}}$ is the transmit signal to noise ratio (SNR). Since imperfect successive interference cancellation (ipSIC) is performed, signal $x_{2}$ is not completely canceled out from the received superposed signal of $U_{1}$ after decoding it, there is residual interference (RI). The received SINR for $U_{1}$ to decode its own signal $x_{1}$ is given by

(4) $$\begin{array}{c} \gamma_{U_{1}}=\frac{a_{1}\rho {\left|{\hat{h}}_{1}\right|}^{2}}{\kappa \rho {\left|{\hat{h}}_{1}\right|}^{2}+\eta_{1}d_{1}^{-\alpha }\rho +1}, \end{array}$$

where κ represents the impact level of RI. $U_{2}$ only needs to treat signal $x_{1}$ of $U_{1}$ as noise to decode its own signal. The received SINR for $U_{2}$ to decode its own signal $x_{2}$ is given by

(5) $$\begin{array}{c} \gamma_{1,U_{2}}=\frac{a_{2}\rho {\left|{\hat{h}}_{0}\right|}^{2}}{a_{1}\rho {\left|{\hat{h}}_{0}\right|}^{2}+\eta_{0}d_{0}^{-\alpha }\rho +1}. \end{array}$$

In the second slot, relaying user $U_{1}$ forwards signal $x_{2}$ decoded from the original superposed signal to $U_{2}$. The received signal at $U_{2}$ for relaying link is written as

(6) $$\begin{array}{c} y_{2,U_{2}}=\left({\hat{h}}_{2}+e_{2}\right)\sqrt{P_{r}}x_{2}+n_{U_{2}}, \end{array}$$

where $P_{r}$ is the normalized transmission power at $U_{1}$. For simplicity, we set $P_{s}=P_{r}=P$. The received SINR for $U_{2}$ to decode signal $x_{2}$ for relaying link is given by

(7) $$\begin{array}{c} \gamma_{2,U_{2}}=\frac{\rho {\left|{\hat{h}}_{2}\right|}^{2}}{\eta_{2}d_{2}^{-\alpha }\rho +1}. \end{array}$$

Hence $U_{2}$ receives signals from two different links of direct link in the first slot and relaying link in the second slot. The total received signal at $U_{2}$ is expressed as

(8) $$\begin{array}{cc} y_{U_{2}} & =\left({\hat{h}}_{0}+e_{0}\right)\left(\sqrt{a_{1}P_{s}}x_{1}+\sqrt{a_{2}P_{s}}x_{2}\right)+\left({\hat{h}}_{2}+e_{2}\right)\sqrt{P_{r}}x_{2}+n_{U_{2}}. \end{array}$$

The received SINR after selection combining (SC) at $U_{2}$ is given by

(9) $$\begin{array}{c} \gamma_{U_{2}}^{SC}=\frac{\rho {\left|{\hat{h}}_{2}\right|}^{2}}{\eta_{2}d_{2}^{-\alpha }\rho +1}+\frac{a_{2}\rho {\left|{\hat{h}}_{0}\right|}^{2}}{a_{1}\rho {\left|{\hat{h}}_{0}\right|}^{2}+\eta_{0}d_{0}^{-\alpha }\rho +1}. \end{array}$$

## 3. Outage Performance Evaluation

In this section, the outage behaviors of downlink cooperative NOMA networks with ipCSI over Nakagami-*m* fading channels are analyzed in the following two scenarios.

### 3.1. User Relaying without Direct Link

In this subsection, the first scenario is investigated in terms of outage probability and diversity order, where the BS communicates with $U_{2}$ via relaying link and $U_{1}$ serves as user relaying to decode and forward the information to $U_{2}$.

#### 3.1.1. Exact Outage Probability

The estimated channel coefficient ${\hat{h}}_{k}$ is subject to Nakagami-*m* distribution, thus the estimated channel gain ${\left|{\hat{h}}_{k}\right|}^{2}$ is subject to Gamma distribution with the fading parameter $m_{k}$ and the estimated link average power ${\hat{\Omega }}_{k}$, k∈{0,1,2}, and its PDF and CDF can be expressed as

(10) $$\begin{array}{c} f_{{\left|{\hat{h}}_{k}\right|}^{2}}\left(x\right)=\frac{m_{k}^{m_{k}}d_{k}^{\alpha m_{k}}x^{m_{k}-1}}{{\left(1-\eta_{k}\right)}^{m_{k}}\Gamma \left(m_{k}\right)}e^{-\frac{m_{k}d_{k}^{\alpha }x}{1-\eta_{k}}}, \end{array}$$

(11) $$\begin{array}{c} F_{{\left|{\hat{h}}_{k}\right|}^{2}}\left(x\right)=\frac{\Upsilon \left(m_{k},m_{k}d_{k}^{\alpha }x/\left(1-\eta_{k}\right)\right)}{\Gamma \left(m_{k}\right)}, \end{array}$$

where $\Gamma \left(\beta \right)=\int_{0}^{\infty }y^{\beta -1}e^{-y}dy$ and $\Upsilon \left(\beta ,y\right)=\int_{0}^{y}y^{\beta -1}e^{-y}dy$ denote the Gamma function and the incomplete Gamma function ([23], eq.(8.310.1), eq.(8.350.1)), respectively. When β takes an integer value greater than or equal to one, we have $\Gamma \left(\beta \right)=\left(\beta -1\right)!$ and $\Upsilon \left(\beta ,y\right)=\left(\beta -1\right)!\left[1-e^{-y}\sum_{l=0}^{\beta -1}\left(y^{l}/l!\right)\right]$ ([23], eq.(8.339.1), eq.(8.352.6)). The physical meaning of Equations (10) and (11) are PDF and CDF that the channels suffer from Nakagami-*m* fading, respectively. Assuming that fading parameter $m_{k}$ is an integer value greater than or equal to one, we can rewrite $f_{{\left|{\hat{h}}_{k}\right|}^{2}}\left(x\right)$ and $F_{{\left|{\hat{h}}_{k}\right|}^{2}}\left(x\right)$ as

(12) $$\begin{array}{c} f_{{\left|{\hat{h}}_{k}\right|}^{2}}\left(x\right)=\frac{m_{k}^{m_{k}}d_{k}^{\alpha m_{k}}x^{m_{k}-1}}{{\left(1-\eta_{k}\right)}^{m_{k}}\left(m_{k}-1\right)!}e^{-\frac{m_{k}d_{k}^{\alpha }x}{1-\eta_{k}}}, \end{array}$$

(13) $$\begin{array}{c} F_{{\left|{\hat{h}}_{k}\right|}^{2}}\left(x\right)=1-e^{-\frac{m_{k}d_{k}^{\alpha }x}{1-\eta_{k}}}\sum_{i=0}^{m_{k}-1}\frac{1}{i!}{\left(\frac{m_{k}d_{k}^{\alpha }x}{1-\eta_{k}}\right)}^{i}. \end{array}$$

In the first scenario, the complementary events of outage occur at $U_{1}$ when $U_{1}$ can successfully decode the signal $x_{2}$ and its own signal $x_{1}$. Based on this explanation, the outage probability of $U_{1}$ can be expressed as

(14) $$\begin{array}{c} P_{U_{1}}=1-\mathrm{Pr}\left(\gamma_{U_{2}\to U_{1}}>\gamma_{th_{2}},\gamma_{U_{1}}>\gamma_{th_{1}}\right), \end{array}$$

where $\gamma_{th_{1}}=2^{2R_{1}}-1$ and $\gamma_{th_{2}}=2^{2R_{2}}-1$ represent the target SNRs at $U_{1}$ to decode $x_{1}$ and $x_{2}$, respectively. $R_{1}$ and $R_{2}$ denote the corresponding target rates.

The exact expression for the outage probability of $U_{1}$ is presented in the following theorem.

**Theorem** **1.** *The closed-form expression for the outage probability of*$U_{1}$*can be derived as*

(15) $$\begin{array}{c} P_{U_{1}}=1-e^{-\delta_{1}\tau }\sum_{j=0}^{m_{1}-1}\frac{{\left(\delta_{1}\tau \right)}^{j}}{j!}, \end{array}$$

*where*$\tau \overset{\Delta }{\;=}\max(\tau_{1},\tau_{2})$*,*$\tau_{1}=\frac{\lambda_{1}\gamma_{th_{1}}}{(a_{1}-\kappa \gamma_{th_{1}})\rho }$*,*$\tau_{2}=\frac{\lambda_{1}\gamma_{th_{2}}}{(a_{2}-a_{1}\gamma_{th_{2}})\rho }$*,*$\lambda_{1}=\eta_{1}d_{1}^{-\alpha }\rho +1$*, and*$\delta_{1}=\frac{m_{1}d_{1}^{\alpha }}{1-\eta_{1}}$*. Note that* (15) *is obtained under the conditions of* $a_{1}>\kappa \gamma_{th_{1}}$ *and* $a_{2}>a_{1}\gamma_{th_{2}}$*. The physical meaning of Equation* (15) *is the probability that the outage occurs for relaying user* $U_{1}$ *over Nakagami-m fading channels in wireless communication networks, in other words, the probability that* $U_{1}$ *fails to decode the signal* $x_{2}$ *of distant user* $U_{2}$ *and/or its own signal* $x_{1}$*.*

**Proof.**  Substituting (3) and (4) into (14), outage probability of $U_{1}$ is calculated as

(16) $$\begin{array}{ll} P_{U_{1}} & =1-\mathrm{Pr}\left({\left|{\hat{h}}_{1}\right|}^{2}>\tau_{2},{\left|{\hat{h}}_{1}\right|}^{2}>\tau_{1}\right)\\ & =1-\mathrm{Pr}\left({\left|{\hat{h}}_{1}\right|}^{2}>\max\left(\tau_{1},\tau_{2}\right)\right)\\ & =1-\mathrm{Pr}\left({\left|{\hat{h}}_{1}\right|}^{2}>\tau \right)\\ & =1-e^{-\delta_{1}\tau }\sum_{j=0}^{m_{1}-1}\frac{{\left(\delta_{1}\tau \right)}^{j}}{j!}, \end{array}$$

(15) can be obtained. The theorem is proved.  □

In the first scenario, the outage events of $U_{2}$ occur if one of the following two events is satisfied. The first event is that $U_{1}$ fails to decode the signal $x_{2}$. The second event is that $U_{2}$ fails to decode its own signal $x_{2}$ when $U_{1}$ can successfully decode the signal $x_{2}$. Based on the above events, the outage probability of $U_{2}$ can be expressed as

(17) $$\begin{array}{cc} P_{U_{2},nodir} & =\mathrm{Pr}\left(\gamma_{U_{2}\to U_{1}}<\gamma_{th_{2}}\right)+\mathrm{Pr}\left(\gamma_{2,U_{2}}<\gamma_{th_{2}},\gamma_{U_{2}\to U_{1}}>\gamma_{th_{2}}\right). \end{array}$$

The exact expression for the outage probability of $U_{2}$ in the first scenario is presented in the following theorem.

**Theorem** **2.** *The closed-form expression for the outage probability of*$U_{2}$*in the first scenario can be derived as*

(18) $$\begin{array}{c} P_{U_{2},nodir}=1-e^{-\left(\delta_{1}\tau_{2}+\delta_{2}\tau_{3}\right)}\sum_{j=0}^{m_{1}-1}\sum_{k=0}^{m_{2}-1}\frac{{\left(\delta_{1}\tau_{2}\right)}^{j}{\left(\delta_{2}\tau_{3}\right)}^{k}}{j!k!}, \end{array}$$

*where*$\tau_{3}=\frac{\lambda_{2}\gamma_{th_{2}}}{\rho }$*,*$\lambda_{2}=\eta_{2}d_{2}^{-\alpha }\rho +1$*, and*$\delta_{2}=\frac{m_{2}d_{2}^{\alpha }}{1-\eta_{2}}$*. The physical meaning of Equation* (18) *is the probability that the outage occurs for distant user* $U_{2}$ *over Nakagami-m fading channels in the first scenario, in other words, the probability that relaying user* $U_{1}$ *fails to decode the signal* $x_{2}$ *of* $U_{2}$ *or* $U_{2}$ *fails to decode its own signal* $x_{2}$ *when* $U_{2}$ *can successfully decode the signal* $x_{2}$*.*

**Proof.**  Substituting (3) and (7) into (17), the outage probability of $U_{2}$ is calculated as

(19) $$\begin{array}{ll} P_{U_{2},nodir} & =\mathrm{Pr}\left({\left|{\hat{h}}_{1}\right|}^{2}<\tau_{2}\right)+\mathrm{Pr}\left({\left|{\hat{h}}_{1}\right|}^{2}>\tau_{2},{\left|{\hat{h}}_{2}\right|}^{2}<\tau_{3}\right)\\ & =\mathrm{Pr}\left({\left|{\hat{h}}_{1}\right|}^{2}<\tau_{2}\right)+\mathrm{Pr}\left({\left|{\hat{h}}_{1}\right|}^{2}>\tau_{2}\right)\mathrm{Pr}\left({\left|{\hat{h}}_{2}\right|}^{2}<\tau_{3}\right)\\ & =1-e^{-\left(\delta_{1}\tau_{2}+\delta_{2}\tau_{3}\right)}\sum_{j=0}^{m_{1}-1}\sum_{k=0}^{m_{2}-1}\frac{{\left(\delta_{1}\tau_{2}\right)}^{j}{\left(\delta_{2}\tau_{3}\right)}^{k}}{j!k!}, \end{array}$$

(18) can be obtained. The theorem is proved.  □

#### 3.1.2. Diversity Analysis

In the first scenario, in order to better understand the outage behavior of the network, the expressions of the approximate outage probability for a pair of NOMA users are derived at high SNR, from which the diversity order achieved by the network can be attained. The diversity order is defined as

(20) $$\begin{array}{c} d=-\lim_{\rho \to \infty }\frac{\log\left(P_{U}^{\infty }\left(\rho \right)\right)}{\log\rho }. \end{array}$$

The physical meaning of Equation (20) is the number of branches that the signal is independently fading in the transmission process, which is shown as the slope of outage probability curve at high SNR.

We rewrite the outage probability expressions of $U_{1}$ and $U_{2}$ as follows:

(21) $$\begin{array}{c} P_{U_{1}}=\mathrm{Pr}\left({\left|{\hat{h}}_{1}\right|}^{2}<\tau \right)=F_{{\left|{\hat{h}}_{1}\right|}^{2}}\left(\tau \right), \end{array}$$

(22) $$\begin{array}{ll} P_{U_{2},nodir} & =\mathrm{Pr}\left({\left|{\hat{h}}_{1}\right|}^{2}<\tau_{2}\right)+\mathrm{Pr}\left({\left|{\hat{h}}_{1}\right|}^{2}>\tau_{2}\right)\mathrm{Pr}\left({\left|{\hat{h}}_{2}\right|}^{2}<\tau_{3}\right)\\ & =F_{{\left|{\hat{h}}_{1}\right|}^{2}}\left(\tau_{2}\right)+\left(1-F_{{\left|{\hat{h}}_{1}\right|}^{2}}\left(\tau_{2}\right)\right)F_{{\left|{\hat{h}}_{2}\right|}^{2}}\left(\tau_{3}\right). \end{array}$$

At high SNR region (ρ→∞), it produces

(23) $$\begin{array}{c} \tau_{1}=\frac{\gamma_{th_{1}}}{a_{1}-\kappa \gamma_{th_{1}}}\left(\eta_{1}d_{1}^{-\alpha }+\frac{1}{\rho }\right)\approx \tau_{1}^{\prime }\eta_{1}d_{1}^{-\alpha }, \end{array}$$

(24) $$\begin{array}{c} \tau_{2}=\frac{\gamma_{th_{2}}}{a_{2}-a_{1}\gamma_{th_{2}}}\left(\eta_{1}d_{1}^{-\alpha }+\frac{1}{\rho }\right)\approx \tau_{2}^{\prime }\eta_{1}d_{1}^{-\alpha }, \end{array}$$

(25) $$\begin{array}{c} \tau =\max\left(\tau_{1}^{\prime },\tau_{2}^{\prime }\right)\left(\eta_{1}d_{1}^{-\alpha }+\frac{1}{\rho }\right)\approx \tau^{\prime }\eta_{1}d_{1}^{-\alpha }, \end{array}$$

(26) $$\begin{array}{c} \tau_{3}=\gamma_{th_{2}}\left(\eta_{2}d_{2}^{-\alpha }+\frac{1}{\rho }\right)\approx \gamma_{th_{2}}\eta_{2}d_{2}^{-\alpha }, \end{array}$$

where $\tau^{\prime }=\max\left(\tau_{1}^{\prime },\tau_{2}^{\prime }\right)$, $\tau_{1}^{\prime }=\frac{\gamma_{th_{1}}}{a_{1}-\kappa \gamma_{th_{1}}}$, and $\tau_{2}^{\prime }=\frac{\gamma_{th_{2}}}{a_{2}-a_{1}\gamma_{th_{2}}}$. Applying the above approximations, we get

(27) $$\begin{array}{c} F_{{\left|{\hat{h}}_{1}\right|}^{2}}\left(\tau_{1}\right)\approx 1-e^{-\chi_{1}{\tau^{\prime }}_{1}}\sum_{j=0}^{m_{1}-1}\frac{{\left(\chi_{1}{\tau^{\prime }}_{1}\right)}^{j}}{j!}, \end{array}$$

(28) $$\begin{array}{c} F_{{\left|{\hat{h}}_{1}\right|}^{2}}\left(\tau_{2}\right)\approx 1-e^{-\chi_{1}{\tau^{\prime }}_{2}}\sum_{j=0}^{m_{1}-1}\frac{{\left(\chi_{1}{\tau^{\prime }}_{2}\right)}^{j}}{j!}, \end{array}$$

(29) $$\begin{array}{c} F_{{\left|{\hat{h}}_{1}\right|}^{2}}\left(\tau \right)\approx 1-e^{-\chi_{1}\tau^{\prime }}\sum_{j=0}^{m_{1}-1}\frac{{\left(\chi_{1}\tau^{\prime }\right)}^{j}}{j!}, \end{array}$$

(30) $$\begin{array}{c} F_{{\left|{\hat{h}}_{2}\right|}^{2}}\left(\tau_{3}\right)\approx 1-e^{-\chi_{2}\gamma_{th_{2}}}\sum_{k=0}^{m_{2}-1}\frac{{\left(\chi_{2}\gamma_{th_{2}}\right)}^{k}}{k!}, \end{array}$$

where $\chi_{1}=\frac{m_{1}\eta_{1}}{1-\eta_{1}}$ and $\chi_{2}=\frac{m_{2}\eta_{2}}{1-\eta_{2}}$. Substituting (27)–(30) into (21) and (22), the approximate outage probabilities of $U_{1}$ and $U_{2}$ at high SNR are obtained as follows

(31) $$\begin{array}{c} P_{U_{1}}^{\infty }=1-e^{-\chi_{1}\tau^{\prime }}\sum_{j=0}^{m_{1}-1}\frac{{\left(\chi_{1}\tau^{\prime }\right)}^{j}}{j!}, \end{array}$$

(32) $$\begin{array}{cc} P_{U_{2},nodir}^{\infty } & =1-e^{-\left(\chi_{1}{\tau^{\prime }}_{2}+\chi_{2}\gamma_{th_{2}}\right)}\sum_{j=0}^{m_{1}-1}\sum_{k=0}^{m_{2}-1}\frac{{\left(\chi_{1}{\tau^{\prime }}_{2}\right)}^{j}{\left(\chi_{2}\gamma_{th_{2}}\right)}^{k}}{j!k!}. \end{array}$$

Substituting (31) and (32) into (20), the diversity orders $d_{U_{1}}$ and $d_{U_{2},nodir}$ achieved by $U_{1}$ and $U_{2}$ are zeros in the first scenario.

From the above analysis, we can observe that $F_{{\left|{\hat{h}}_{1}\right|}^{2}}\left(\tau_{1}\right)$, $F_{{\left|{\hat{h}}_{1}\right|}^{2}}\left(\tau_{2}\right)$, $F_{{\left|{\hat{h}}_{1}\right|}^{2}}\left(\tau \right)$, and $F_{{\left|{\hat{h}}_{2}\right|}^{2}}\left(\tau_{3}\right)$ maintain constant with the increase of $d_{1}$ and $d_{2}$ when ρ→∞. Hence an error floor appears in the outage probability owing to the existence of channel estimation errors under ipCSI conditions even though the transmit SNR is extremely high. It is worth noting that the error floor results in the diversity order to be zero at high SNR. Hence the error floors for $P_{U_{1}}$ and $P_{U_{2},nodir}$ are $P_{U_{1}}^{\infty }$ and $P_{U_{2},nodir}^{\infty }$, respectively, which are independent of $d_{1}$ and $d_{2}$.

### 3.2. User Relaying with Direct Link

In this subsection, we investigate another representative scenario, where the BS communicates with $U_{2}$ by way of relaying link and direct link. As a result, $U_{2}$ receives the signals from two different paths and the reliability of the signal received by $U_{2}$ has been improved. Since the direct link has no effect on $U_{1}$, we only investigate the outage performance of $U_{2}$.

#### 3.2.1. Exact Outage Probability

In the second scenario, the outage events of $U_{2}$ occur when one of the following two events happens. The first event is that $U_{1}$ can successfully decode the signal $x_{2}$, but the received SINR of $U_{2}$ after SC cannot meet its target SNR. The second event is that both $U_{1}$ and $U_{2}$ fail to decode the signal $x_{2}$. Based on the above events, the outage probability of $U_{2}$ can be expressed as

(33) $$\begin{array}{cc} P_{U_{2},dir} & =\mathrm{Pr}\left(\gamma_{U_{2}}^{SC}<\gamma_{th_{2}},\gamma_{U_{2}\to U_{1}}>\gamma_{th_{2}}\right)+\mathrm{Pr}\left(\gamma_{U_{2}\to U_{1}}<\gamma_{th_{2}},\gamma_{1,U_{2}}<\gamma_{th_{2}}\right). \end{array}$$

The exact expression for the outage probability of $U_{2}$ in the second scenario is presented in the following theorem.

**Theorem** **3.** *The closed-form expression for the outage probability of*$U_{2}$*in the second scenario can be derived as*

(34) PU2,dir=1−e−δ0τ4∑i=0m0−1δ0τ4ii!−∑δ0m0+nδ1τ2jδ2kkrrsm0−1t(−1)n+r+s+m0−t−1j!k!n!(m0−1)!τ3k−reμ−δ1τ2×πrωs+m0−t−1(−1)N+2φN+1(N+1)!Ei(ψ1)−Ei(ψ2)+∑m=0Neψ1ψ1m(τ4+ω)N+1−eψ2ψ2mωN+1(N+1)N⋯(N+1−m),

*where*$\sum =\sum_{j=0}^{m_{1}-1}\sum_{k=0}^{m_{2}-1}\sum_{r=0}^{k}\sum_{s=0}^{r}\sum_{t=0}^{m_{0}-1}\sum_{n=0}^{\infty }$*,*$\delta_{0}=\frac{m_{0}d_{0}^{\alpha }}{1-\eta_{0}}$*,*$\delta_{1}=\frac{m_{1}d_{1}^{\alpha }}{1-\eta_{1}}$*,*$\delta_{2}=\frac{m_{2}d_{2}^{\alpha }}{1-\eta_{2}}$*,*$\tau_{2}=\frac{\lambda_{1}\gamma_{th_{2}}}{(a_{2}-a_{1}\gamma_{th_{2}})\rho }$*,*$\tau_{3}=\frac{\lambda_{2}\gamma_{th_{2}}}{\rho }$*,*$\tau_{4}=\frac{\lambda_{0}\gamma_{th_{2}}}{(a_{2}-a_{1}\gamma_{th_{2}})\rho }$*,*$\lambda_{0}=\eta_{0}d_{0}^{-\alpha }\rho +1$*,*$\lambda_{1}=\eta_{1}d_{1}^{-\alpha }\rho +1$*,*$\lambda_{2}=\eta_{2}d_{2}^{-\alpha }\rho +1$*,*$\omega =\frac{\lambda_{0}}{a_{1}\rho }$*,*$\pi =\frac{a_{2}\lambda_{2}}{a_{1}\rho }$*,*$\mu =\delta_{0}\omega +\delta_{2}\pi -\delta_{2}\tau_{3}$*,*$\varphi =\delta_{2}\pi \omega$*,*N=n+t−s*,*$\psi_{1}=\frac{-\delta_{2}\pi \lambda_{0}}{a_{1}\rho \tau_{4}+\lambda_{0}}$*, and*$\psi_{2}=-\delta_{2}\pi$*.*Ei(·)*is the exponential integral function ([23], eq.(8.211.1)). The physical meaning of Equation* (34) *is the probability that the outage occurs for distant user* $U_{2}$ *over Nakagami-m fading channels in the second scenario, in other words, the probability that relaying user* $U_{1}$ *can successfully decode the signal* $x_{2}$ *of* $U_{2}$*, but the received SINR of* $U_{2}$ *after SC cannot meet its target SNR or both* $U_{1}$ *and* $U_{2}$ *fail to decode the signal* $x_{2}$*.*

**Proof.**  See Appendix A.  □

#### 3.2.2. Diversity Analysis

In the second scenario, the approximate outage probability of $U_{2}$ is derived at high SNR, and the diversity order achieved by $U_{2}$ is analyzed.

We define the three probabilities based on (33) by $\Phi_{1}$, $\Phi_{2}$, and $\Phi_{3}$, respectively, and rewrite them as follows

(35) $$\begin{array}{ll} \Phi_{1} & =\mathrm{Pr}\left({\left|{\hat{h}}_{2}\right|}^{2}<\tau_{3}-\frac{a_{2}\lambda_{2}{\left|{\hat{h}}_{0}\right|}^{2}}{a_{1}\rho {\left|{\hat{h}}_{0}\right|}^{2}+\lambda_{0}},{\left|{\hat{h}}_{0}\right|}^{2}<\tau_{4}\right)\\ & =\int_{0}^{\tau_{4}}F_{{\left|{\hat{h}}_{2}\right|}^{2}}\left(\tau_{3}-\frac{a_{2}\lambda_{2}x}{a_{1}\rho x+\lambda_{0}}\right)f_{{\left|{\hat{h}}_{0}\right|}^{2}}\left(x\right)dx, \end{array}$$

(36) $$\begin{array}{c} \Phi_{2}=1-\mathrm{Pr}\left({\left|{\hat{h}}_{1}\right|}^{2}<\tau_{2}\right)=1-F_{{\left|{\hat{h}}_{1}\right|}^{2}}\left(\tau_{2}\right), \end{array}$$

(37) $$\begin{array}{cc} \Phi_{3} & =\mathrm{Pr}\left({\left|{\hat{h}}_{0}\right|}^{2}<\tau_{4}\right)\mathrm{Pr}\left({\left|{\hat{h}}_{1}\right|}^{2}<\tau_{2}\right)=F_{{\left|{\hat{h}}_{0}\right|}^{2}}\left(\tau_{4}\right)F_{{\left|{\hat{h}}_{1}\right|}^{2}}\left(\tau_{2}\right). \end{array}$$

When ρ→∞, it yields

(38) $$\begin{array}{cc} \tau_{3}-\frac{a_{2}\lambda_{2}x}{a_{1}\rho x+\lambda_{0}} & =\gamma_{th_{2}}\left(\eta_{2}d_{2}^{-\alpha }+\frac{1}{\rho }\right)-\frac{a_{2}x\left(\eta_{2}d_{2}^{-\alpha }+\frac{1}{\rho }\right)}{a_{1}x+\eta_{0}d_{0}^{-\alpha }+\frac{1}{\rho }}\approx \gamma_{th_{2}}\eta_{2}d_{2}^{-\alpha }-\frac{a_{2}\eta_{2}d_{2}^{-\alpha }x}{a_{1}x+\eta_{0}d_{0}^{-\alpha }}, \end{array}$$

and

(39) $$\begin{array}{c} \tau_{4}=\frac{\gamma_{th_{2}}}{a_{2}-a_{1}\gamma_{th_{2}}}\left(\eta_{0}d_{0}^{-\alpha }+\frac{1}{\rho }\right)\approx \tau_{4}^{\prime }\eta_{0}d_{0}^{-\alpha }, \end{array}$$

respectively, where $\tau_{2}^{\prime }=\tau_{4}^{\prime }=\frac{\gamma_{th_{2}}}{a_{2}-a_{1}\gamma_{th_{2}}}$. Using the above approximations, we have

(40) $$\begin{array}{cc} F_{{\left|{\hat{h}}_{2}\right|}^{2}}\left(\tau_{3}-\frac{a_{2}\lambda_{2}x}{a_{1}\rho x+\lambda_{0}}\right) & \approx 1-e^{-\delta_{2}\left(\gamma_{th_{2}}\eta_{2}d_{2}^{-\alpha }-\frac{a_{2}\eta_{2}d_{2}^{-\alpha }x}{a_{1}x+\eta_{0}d_{0}^{-\alpha }}\right)}\sum_{k=0}^{m_{2}-1}\frac{\delta_{2}^{k}}{k!}{\left(\gamma_{th_{2}}\eta_{2}d_{2}^{-\alpha }-\frac{a_{2}\eta_{2}d_{2}^{-\alpha }x}{a_{1}x+\eta_{0}d_{0}^{-\alpha }}\right)}^{k}, \end{array}$$

and

(41) $$\begin{array}{c} F_{{\left|{\hat{h}}_{0}\right|}^{2}}\left(\tau_{4}\right)\approx 1-e^{-\chi_{0}{\tau^{\prime }}_{4}}\sum_{i=0}^{m_{0}-1}\frac{{\left(\chi_{0}{\tau^{\prime }}_{4}\right)}^{i}}{i!}, \end{array}$$

respectively, where $\chi_{0}=\frac{m_{0}\eta_{0}}{1-\eta_{0}}$.

Substituting (12), (39), and (40) into (35), using ([23], eq.(3.351.1)) and the Binomial theorem, the approximation of $\Phi_{1}$ can be given by

(42) Φ1≈∫0τ′4η0d0−αFh^22γth2η2d2−α−a2η2d2−αxa1x+η0d0−αδ0m0xm0−1(m0−1)!e−δ0xdx=∫0εδ0m0xm0−1(m0−1)!e−δ0xdx−δ0m0e−δ2ν(m0−1)!∫0εeδ2a2η2d2−αxa1x+η0d0−α∑k=0m2−1δ2kk!ν−a2η2d2−αxa1x+η0d0−αkxm0−1e−δ0xdx=1−e−δ0ε∑i=0m0−1δ0εii!−δ0m0e−δ2ν(m0−1)!∑k=0m2−1∑r=0k(−1)rk!krδ2kνk−rυr∫0εxx+ϖrxm0−1eδ2υxx+ϖe−δ0xdx,

where $\nu =\gamma_{th_{2}}\eta_{2}d_{2}^{-\alpha }$, $\varepsilon =\tau_{4}^{\prime }\eta_{0}d_{0}^{-\alpha }$, $\upsilon =\frac{a_{2}\eta_{2}d_{2}^{-\alpha }}{a_{1}}$, and $\varpi =\frac{\eta_{0}d_{0}^{-\alpha }}{a_{1}}$.

Using y=x+ϖ, applying the Binomial theorem and power series, the integration on the right side of (42) can be written as

(43) Ξ1=∫0εxx+ϖrxm0−1eδ2υxx+ϖe−δ0xdx=eδ0ϖ+δ2υ∫ϖε+ϖ1−ϖyry−ϖm0−1e−ϕye−δ0ydy=eδ0ϖ+δ2υ∑s=0r∑t=0m0−1∑n=0∞(−1)n+s+m0−t−1n!ϖs+m0−t−1rsm0−1tδ0n∫ϖε+ϖyn+t−se−ϕydy,

where $\phi =\delta_{2}\upsilon \varpi$.

Using $y=\frac{1}{z}$ and ([23], eq. (3.351.4)), the integration on the right side of (43) can be calculated as

(44) $$\begin{array}{ll} \Xi_{2} & =\int_{\varpi }^{\varepsilon +\varpi }y^{n+t-s}e^{-\frac{\phi }{y}}dy\\ & =\int_{\frac{1}{\varepsilon +\varpi }}^{\frac{1}{\varpi }}\frac{1}{z^{n+t-s+2}}e^{-\phi z}dz\\ & =\frac{{\left(-1\right)}^{N+2}\phi^{N+1}}{(N+1)!}\left(\mathrm{Ei}\left(\Psi_{1}\right)-\mathrm{Ei}\left(\Psi_{2}\right)\right)+\sum_{m=0}^{N}\frac{e^{\Psi_{1}}\Psi_{1}^{m}{\left(\varepsilon +\varpi \right)}^{N+1}-e^{\Psi_{2}}\Psi_{2}^{m}\varpi^{N+1}}{\left(N+1)N\cdots (N+1-m\right)}, \end{array}$$

where N=n+t−s, $\Psi_{1}=\frac{-\phi }{\varepsilon +\varpi }$, and $\Psi_{2}=-\delta_{2}\upsilon$.

Substituting (43) into (42), the approximation of $\Phi_{1}$ can be written as

(45) Φ1≈1−e−δ0ε∑i=0m0−1δ0εii!−∑k=0m2−1∑r=0k∑s=0r∑t=0m0−1∑n=0∞krrsm0−1t(−1)n+r+s+m0−t−1k!n!(m0−1)!δ0m0+nδ2kνk−r×υreθϖs+m0−t−1(−1)N+2ϕN+1(N+1)!Ei(Ψ1)−Ei(Ψ2)+∑m=0NeΨ1Ψ1m(ε+ϖ)N+1−eΨ2Ψ2mϖN+1(N+1)N⋯(N+1−m),

where $\theta =\delta_{0}\varpi +\delta_{2}\upsilon -\delta_{2}\nu$.

We substitute (24), (28), (39), and (41) into (36) and (37), the approximations of $\Phi_{2}$ and $\Phi_{3}$ can be calculated as

(46) $$\begin{array}{c} \Phi_{2}\approx 1-F_{{\left|{\hat{h}}_{1}\right|}^{2}}\left(\tau_{2}^{\prime }\eta_{1}d_{1}^{-\alpha }\right)=e^{-\delta_{1}\xi }\sum_{j=0}^{m_{1}-1}\frac{{\left(\delta_{1}\xi \right)}^{j}}{j!}, \end{array}$$

(47) $$\begin{array}{cc} \Phi_{3} & \approx F_{{\left|{\hat{h}}_{0}\right|}^{2}}\left({\tau^{\prime }}_{4}\eta_{0}d_{0}^{-\alpha }\right)F_{{\left|{\hat{h}}_{1}\right|}^{2}}\left({\tau^{\prime }}_{2}\eta_{1}d_{1}^{-\alpha }\right)\\ & =1-e^{-\delta_{0}\varepsilon }\sum_{i=0}^{m_{0}-1}\frac{{\left(\delta_{0}\varepsilon \right)}^{i}}{i!}-e^{-\delta_{1}\xi }\sum_{j=0}^{m_{1}-1}\frac{{\left(\delta_{1}\xi \right)}^{j}}{j!}+e^{-\left(\delta_{0}\varepsilon +\delta_{1}\xi \right)}\sum_{i=0}^{m_{0}-1}\sum_{j=0}^{m_{1}-1}\frac{{\left(\delta_{0}\varepsilon \right)}^{i}{\left(\delta_{1}\xi \right)}^{j}}{i!j!}, \end{array}$$

where $\xi =\tau_{2}^{\prime }\eta_{1}d_{1}^{-\alpha }$.

Equations (45)–(47) are substituted into (33), the approximate outage probability of $U_{2}$ at high SNR is obtained as follows

(48) PU2,dir∞=Φ1∞Φ2∞+Φ3∞=1−e−δ0ε∑i=0m0−1δ0εii!−∑krrsm0−1t(−1)n+r+s+m0−t−1j!k!n!(m0−1)!δ0m0+nδ1ξjδ2kνk−rυreθ−δ1ξ×ϖs+m0−t−1(−1)N+2ϕN+1(N+1)!(Ei(Ψ1)−Ei(Ψ2))+∑m=0NeΨ1Ψ1m(ε+ϖ)N+1−eΨ2Ψ2mϖN+1(N+1)N⋯(N+1−m),

where $\sum =\sum_{j=0}^{m_{1}-1}\sum_{k=0}^{m_{2}-1}\sum_{r=0}^{k}\sum_{s=0}^{r}\sum_{t=0}^{m_{0}-1}\sum_{n=0}^{\infty }$. Substituting (48) into (20), the diversity order $d_{U_{2},dir}$ achieved by $U_{2}$ is zero in the second scenario.

Similar to the first scenario, $F_{{\left|{\hat{h}}_{0}\right|}^{2}}\left(\tau_{4}\right)$, $F_{{\left|{\hat{h}}_{1}\right|}^{2}}\left(\tau_{2}\right)$, and $F_{{\left|{\hat{h}}_{2}\right|}^{2}}\left(\tau_{3}-\frac{a_{2}\lambda_{2}x}{a_{1}\rho x+\lambda_{0}}\right)$ remain unchanged as $d_{1}$ and $d_{2}$ increase when ρ→∞. Thus there is the error floor for outage probability as a result of channel estimation errors under ipCSI conditions even if ρ is very large, from which the diversity order achieved by $U_{2}$ is zero at high SNR. Hence the error floor for $P_{U_{2},dir}$ is $P_{U_{2},dir}^{\infty }$, which is independent of $d_{1}$ and $d_{2}$.

## 4. Numerical Results

In this section, numerical results are presented to evaluate the outage performance of cooperative NOMA networks with ipCSI in terms of outage probability over Nakagami-*m* fading channels. We use MATLAB programming software for simulation by setting reasonable parameters. The exact expressions for the outage probability are verified by utilizing Monte Carlo simulations. In addition, OMA is regarded as the benchmark to compare with NOMA. Considering that the BS, $U_{1}$, and $U_{2}$ are located in a straight line. Without loss of generality, assuming that the distance between the BS and $U_{2}$ is normalized to unity, i.e., $d_{0}=1$, and we can obtain $d_{2}=1-d_{1}$, where $d_{1}$ and $d_{2}$ are the normalized distance between the BS and $U_{1}$, and between $U_{1}$ and $U_{2}$, respectively. In the following simulations, we set the simulation parameters in Table 3.

**Figure 2:**
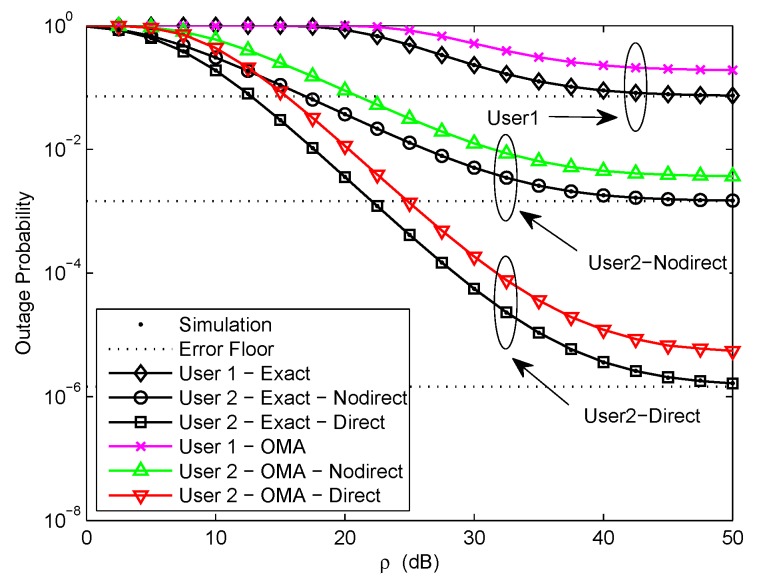
Outage probability versus transmit signal–noise–ratio (SNR).

Figure 2 plots the outage probability of a pair of users for the two scenarios versus the transmit SNR. We assume κ=0.0001, η=0.00001, and $d_{1}=0.5$. The target rate is set to be $R_{1}=3.6$, $R_{2}=1$ bit per channel use (BPCU) for $U_{1}$ and $U_{2}$, respectively. The exact outage probability curves of a pair of users for the two scenarios are plotted according to (15), (18), and (34), respectively. We can easily observe that the exact outage probability curves and the Monte Carlo simulation results match well. It can be seen that the outage performance of NOMA outperforms OMA. It is the fact that the superposition coding scheme is performed at the transmitter in NOMA networks, multiple users can be served by sharing the same physical resource. To ensure user fairness, the target rate of OMA user is larger than that of NOMA user. The approximate outage probability curves of a pair of users for the two scenarios are plotted according to (31), (32), and (48), respectively. It is observed that the outage probability decreases as the transmit SNR increases at low SNR and reaches a fixed value at high SNR. The error floor exists at high SNR owing to the channel estimation errors, which leads zero diversity order. Another important observation is that the outage probability of $U_{2}$ with direct link in the second scenario is much better than that of $U_{2}$ without direct link in the first scenario and the error floor gap is about 3 orders of magnitude. Because $U_{2}$ only receives the signal from relaying link in the first scenario, but $U_{2}$ receives the signals from relaying link and direct link in the second scenario, thus the reliability of the signal received by $U_{2}$ in the second scenario has been improved.

**Figure 3:**
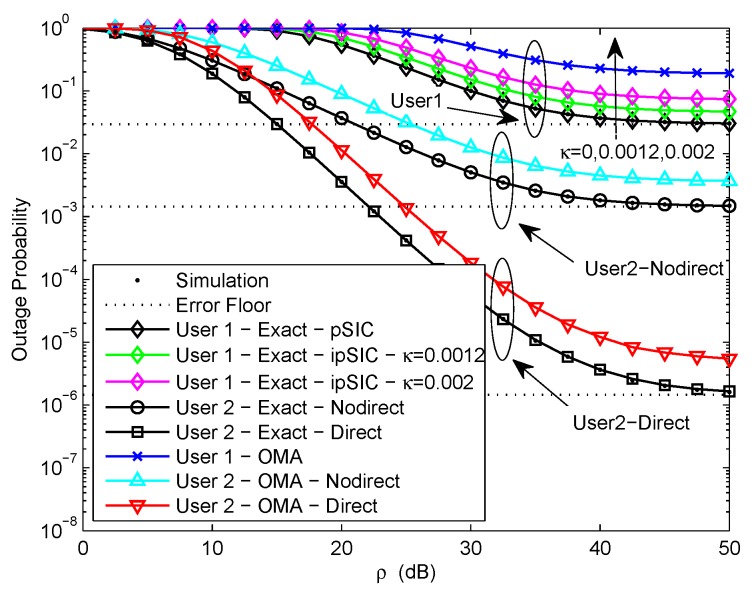
Outage probability versus transmit SNR with different levels of RI.

Figure 3 plots the outage probability of a pair of users for the two scenarios versus the transmit SNR with different levels of RI from 0 to 0.002. We assume $R_{1}=3$, $R_{2}=1$, η=0.0001, and $d_{1}=0.5$. Obviously, the exact outage probability curves match perfectly with the Monte Carlo simulation results. We observe that NOMA is capable of achieving better outage performance than OMA. This is caused by the superposition coding scheme. Considering the impact of RI caused by ipSIC at user $U_{1}$, the outage probability of $U_{1}$ with different levels of RI is plotted based on (15). It can be observed that the RI-based exact outage probability curves of $U_{1}$ reduce with the increase of the transmit SNR in low SNR region and an error floor appears in high SNR region. This is due to the existence of channel estimation errors, resulting in zero diversity order. More importantly, it is shown that the effect of RI on the outage performance of $U_{1}$ is very obvious. The outage performance of $U_{1}$ reduce significantly increasing the levels of RI from 0 to 0.002. This is because the larger the levels of RI, the greater the interference of $U_{1}$, hence the outage performance of $U_{1}$ becomes worse. Therefore, it is extremely important to consider the effect of RI in practical ipSIC systems.

**Figure 4:**
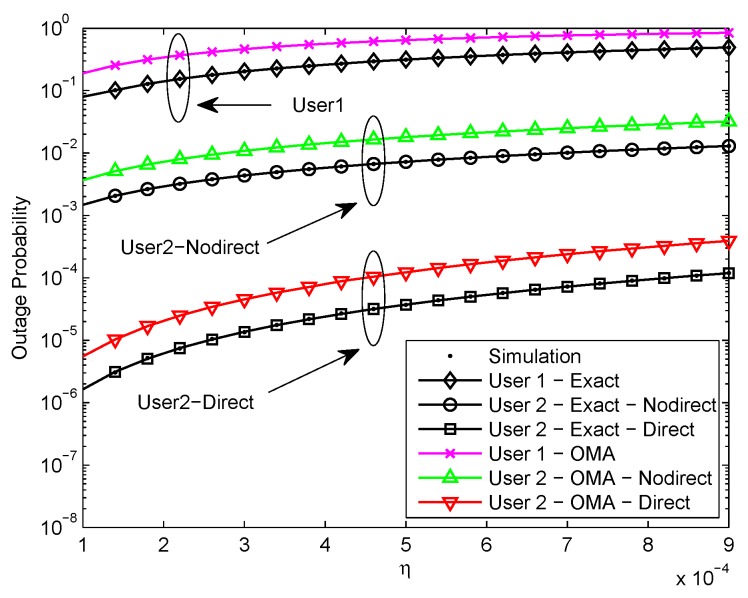
Outage probability versus relative channel estimation error.

Figure 4 plots the outage probability of a pair of users for the two scenarios versus the relative channel estimation error. We assume $R_{1}=3.6$, $R_{2}=1$, κ=0.0001, ρ=50 dB, and $d_{1}=0.5$. We can see that the exact outage probability curves and the Monte Carlo simulation results are in excellent agreement. One can observe that the outage performance of NOMA is superior to OMA. It is due to the superposition coding scheme. Moreover, it is observed that the outage probability increases as the relative channel estimation error increases due to the impact of error floor. In addition, it is worth noting that the outage performance of $U_{2}$ with direct link in the second scenario exceeds $U_{2}$ without direct link in the first scenario and the outage performance gap is about 3 orders of magnitude.It is that $U_{2}$ only receives the signal from relaying link in the first scenario, but $U_{2}$ receives the signals from two different paths in the second scenario, thus the performance of $U_{2}$ in the second scenario is much better.

**Figure 5:**
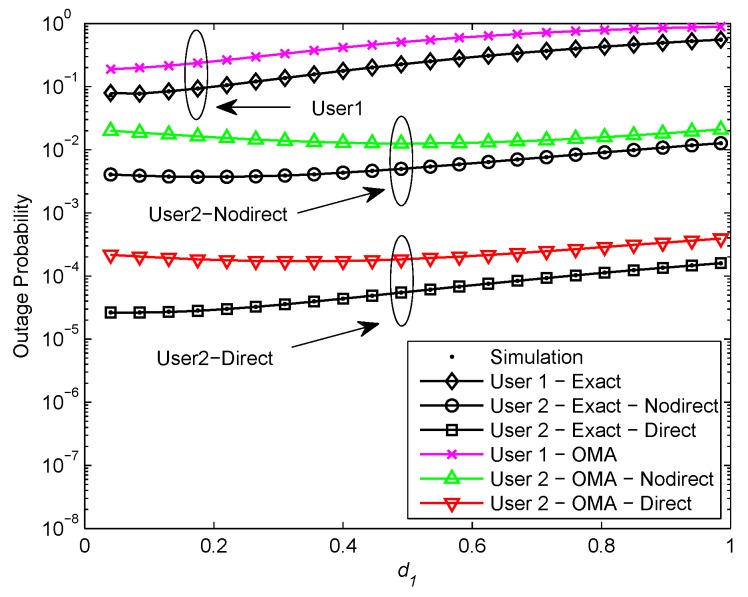
Outage probability versus normalized distance between BS and $U_{1}$ (ρ = 30 dB).

**Figure 6:**
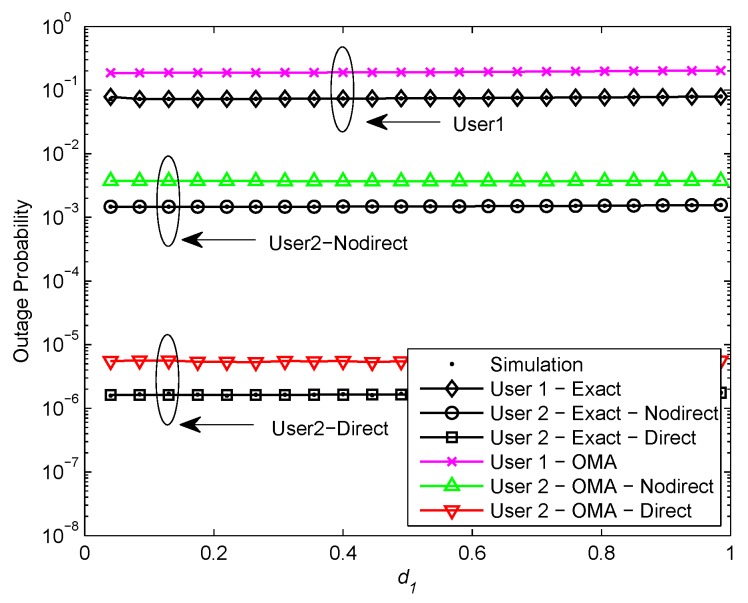
Outage probability versus normalized distance between BS and $U_{1}$ (ρ = 50 dB).

Figure 5 and Figure 6 plot the outage probability of a pair of users for the two scenarios versus the normalized distance between BS and $U_{1}$ for ρ=30 and ρ=50 dB, respectively. We assume $R_{1}=3.6$, $R_{2}=1$, κ=0.0001, and η=0.0001. In Figure 5, it is shown that the exact outage probability curves match precisely with the Monte Carlo simulation results. We observe that the optimal location for user relaying $U_{1}$ is closer to the BS than $U_{2}$. The reason is that $U_{1}$ with better channel condition is allocated less transmit power, the optimal location for $U_{1}$ should be nearer to the BS in order to achieve high received SNR at $U_{1}$. Furthermore, it can be observed that the outage performance of NOMA exceeds OMA. That is owing to the superposition coding scheme. It is worth pointing out that the outage performance declines as $U_{1}$ gets close to $U_{2}$ and the outage performance gap between NOMA and OMA is no longer apparent. It is the fact that $U_{1}$ with better channel condition is allocated less transmit power, the received SNR at $U_{1}$ reduces as $U_{1}$ gets close to $U_{2}$. Therefore, the user relaying location for cooperative NOMA networks should be near to the BS. Additionally, it is observed that the outage performance of $U_{2}$ with direct link in the second scenario outperforms $U_{2}$ without direct link in the first scenario and the outage performance gap is about 2 orders of magnitude. Since $U_{2}$ in the second scenario has more paths to receive signals than $U_{2}$ in the first scenario. In Figure 6, it can be seen that the outage probability maintains constant as the user relaying location increases. This phenomenon can be explained that the outage probability achieves the error floor at high SNR which is independent of $d_{1}$ and $d_{2}$.

## 5. Conclusions

This paper has investigated the downlink cooperative NOMA network with ipCSI over Nakagami-*m* fading channels. The outage performance of two cooperative relaying scenarios is analyzed in detail. We derive the closed-form expressions for the exact outage probability to characterize the outage behavior of the network. Then the expressions for the approximate outage probability at high SNR are derived, from which the diversity order achieved by the network is zero due to the effect of channel estimation errors. Simulation results demonstrate that NOMA is superior to OMA in terms of outage probability. It can be seen that an error floor appears in the outage probability at high SNR. Furthermore, the optimal user relaying location for cooperative NOMA networks should be close to the BS. The outage performance of the distant user can be greatly improved by using the direct link between the BS and distant user. Our future work will relax the assumption of half-duplex mode, impact of loop interference on system performance will be investigated under ipCSI in full-duplex mode.

## Figures and Tables

**Table 1:** The comparison of non-orthogonal multiple access (NOMA) and conventional orthogonal multiple access (OMA).

Multiple Access Scheme	NOMA	Conventional OMA
Aim	Higher spectral efficiency, massive connectivity and user fairness.	Good system throughput, low cost of receiver.
Solution	Superposition coding scheme at the transmitter, successive interference cancellation at the receiver and more transmit power is allocated to users with worse channel conditions.	Multiple users are allocated with radio resources which are orthogonal in time, frequency, or code domain.
Pros	Multiple users can be served by sharing the same physical resource. The number of supported users or devices is not strictly limited by the amount of available resources and their scheduling granularity.	No interference exists among multiple users. Low complexity of receiver.
Cons	Interference exists among multiple users. High complexity of receiver.	A single radio resource can only be allocated to a user. The maximum number of supported users is limited by the total amount and the scheduling granularity of orthogonal resources.

**Table 2:** The main notations used in this paper.

Notation	Description
Pr[·]	Probability
$f_{X}\left(\cdot \right)$	Probability density function (PDF) of random variable *X*
$F_{X}\left(\cdot \right)$	Cumulative distribution function (CDF) of random variable *X*
$E\left\{\cdot \right\}$	Expectation operator
$\mathrm{CN}(0,\sigma_{e_{k}}^{2})$	Circularly symmetric complex Gaussian distribution $e_{k}$ with mean zero and variance $\sigma_{e_{k}}^{2}$
$m_{k}$	Fading parameter of channel *k*
$d_{k}$	Distance between two nodes of channel *k*
α	Path loss exponent
$\eta_{k}$	Relative channel estimation error of channel *k*
ρ	Transmit signal to noise ratio (SNR)
κ	Impact level of residual interference (RI)
$R_{1}$	Target rate of user $U_{1}$
$R_{2}$	Target rate of user $U_{2}$
$\gamma_{th_{1}}$	Target SNR of user $U_{1}$
$\gamma_{th_{2}}$	Target SNR of user $U_{2}$

**Table 3:** The simulation parameters.

Description	NOMA	OMA
Power allocation coefficient	$a_{1}=0.2$, $a_{2}=0.8$	
Path loss exponent	α=2	
Fading parameter	$m_{0}=m_{1}=m_{2}=1$	
Relative channel estimation error	$\eta_{0}=\eta_{1}=\eta_{2}=1\times 10^{-4}∼9\times 10^{-4}$	
Distance between two nodes	$d_{0}=1$, $d_{1}=0.04∼1$, $d_{2}=1-d_{1}$	
Impact level of RI	κ=0,0.0001,0.0012,0.002	
Transmit SNR	ρ=0∼50dB	
Target rate	$R_{1}=3,3.6\mathrm{BPCU}$, $R_{2}=1\mathrm{BPCU}$	$R_{1}=5.5\mathrm{BPCU}$, $R_{2}=2.1\mathrm{BPCU}$

## Appendix A

**Proof** **of** **Theorem 3.** According to (33), the outage probability of $U_{2}$ in the second scenario is expressed as

(A1) $$\begin{array}{cc} P_{U_{2},dir} & =\underset{\Phi_{1}}{\underset{︸}{\mathrm{Pr}(\gamma_{U_{2}}^{SC}<\gamma_{th_{2}})}}\underset{\Phi_{2}}{\underset{︸}{\mathrm{Pr}(\gamma_{U_{2}\to U_{1}}>\gamma_{th_{2}})}}+\underset{\Phi_{3}}{\underset{︸}{\mathrm{Pr}(\gamma_{U_{2}\to U_{1}}<\gamma_{th_{2}},\gamma_{1,U_{2}}<\gamma_{th_{2}})}}. \end{array}$$

Substituting (3), (5), and (9) into (A1), $\Phi_{1}$, $\Phi_{2}$, and $\Phi_{3}$ can be obtained as follows

(A2) $$\begin{array}{cc} \Phi_{1} & =\mathrm{Pr}\left({\left|{\hat{h}}_{2}\right|}^{2}<\tau_{3}-\frac{a_{2}\lambda_{2}{\left|{\hat{h}}_{0}\right|}^{2}}{a_{1}\rho {\left|{\hat{h}}_{0}\right|}^{2}+\lambda_{0}},{\left|{\hat{h}}_{0}\right|}^{2}<\tau_{4}\right)\\ & =\int_{0}^{\tau_{4}}F_{{\left|{\hat{h}}_{2}\right|}^{2}}\left(\tau_{3}-\frac{a_{2}\lambda_{2}x}{a_{1}\rho x+\lambda_{0}}\right)f_{{\left|{\hat{h}}_{0}\right|}^{2}}\left(x\right)dx\\ & =\int_{0}^{\tau_{4}}\frac{\delta_{0}^{m_{0}}x^{m_{0}-1}}{(m_{0}-1)!}e^{-\delta_{0}x}dx-\frac{\delta_{0}^{m_{0}}e^{-\delta_{2}\tau_{3}}}{(m_{0}-1)!}\int_{0}^{\tau_{4}}e^{\frac{\delta_{2}a_{2}\lambda_{2}x}{a_{1}\rho x+\lambda_{0}}}\sum_{k=0}^{m_{2}-1}\frac{\delta_{2}^{k}}{k!}{\left(\tau_{3}-\frac{a_{2}\lambda_{2}x}{a_{1}\rho x+\lambda_{0}}\right)}^{k}x^{m_{0}-1}e^{-\delta_{0}x}dx, \end{array}$$

where $\delta_{0}=\frac{m_{0}d_{0}^{\alpha }}{1-\eta_{0}}$, $\delta_{2}=\frac{m_{2}d_{2}^{\alpha }}{1-\eta_{2}}$, $\tau_{3}=\frac{\lambda_{2}\gamma_{th_{2}}}{\rho }$, $\tau_{4}=\frac{\lambda_{0}\gamma_{th_{2}}}{(a_{2}-a_{1}\gamma_{th_{2}})\rho }$, $\lambda_{0}=\eta_{0}d_{0}^{-\alpha }\rho +1$, and $\lambda_{2}=\eta_{2}d_{2}^{-\alpha }\rho +1$. Using ([23], eq.(3.351.1)) and the Binomial theorem, $\Phi_{1}$ can be given by

(A3) Φ1=1−e−δ0τ4∑i=0m0−1δ0τ4ii!−δ0m0e−δ2τ3(m0−1)!∑k=0m2−1∑r=0kkr(−1)rk!δ2kτ3k−rπr×∫0τ4xx+ωrxm0−1eδ2πxx+ωe−δ0xdx︸Θ1,

where $\omega =\frac{\lambda_{0}}{a_{1}\rho }$ and $\pi =\frac{a_{2}\lambda_{2}}{a_{1}\rho }$. Furthermore, using y=x+ω, $\Theta_{1}$ can be calculated as

(A4) $$\begin{array}{c} \Theta_{1}=e^{\delta_{0}\omega +\delta_{2}\pi }\underset{\Theta_{2}}{\underset{︸}{\int_{\omega }^{\tau_{4}+\omega }{\left(1-\frac{\omega }{y}\right)}^{r}{\left(y-\omega \right)}^{m_{0}-1}e^{-\frac{\varphi }{y}}e^{-\delta_{0}y}dy}}, \end{array}$$

where $\varphi =\delta_{2}\pi \omega$. Applying the Binomial theorem and power series, $\Theta_{2}$ can be written as

(A5) Θ2=∫ωτ4+ω∑s=0rrs(−1)sωys∑t=0m0−1(−1)m0−t−1m0−1tωm0−t−1yte−φy∑n=0∞−δ0ynn!dy=∑s=0r∑t=0m0−1∑n=0∞(−1)n+s+m0−t−1n!ωs+m0−t−1δ0nrsm0−1t∫ωτ4+ωyn+t−se−φydy︸Θ3.

Using $y=\frac{1}{z}$ and [23, eq.(3.351.4)], $\Theta_{3}$ is calculated as

(A6) $$\begin{array}{cc} \Theta_{3} & =\int_{\frac{1}{\tau_{4}+\omega }}^{\frac{1}{\omega }}\frac{1}{z^{n+t-s+2}}e^{-\varphi z}dz\\ & =\frac{{\left(-1\right)}^{N+2}\varphi^{N+1}}{(N+1)!}\left(\mathrm{Ei}\left(\psi_{1}\right)-\mathrm{Ei}\left(\psi_{2}\right)\right)+\sum_{m=0}^{N}\frac{e^{\psi_{1}}\psi_{1}^{m}{\left(\tau_{4}+\omega \right)}^{N+1}-e^{\psi_{2}}\psi_{2}^{m}\omega^{N+1}}{\left(N+1)N\cdots (N+1-m\right)}, \end{array}$$

where N=n+t−s, $\psi_{1}=\frac{-\delta_{2}\pi \lambda_{0}}{a_{1}\rho \tau_{4}+\lambda_{0}}$, and $\psi_{2}=-\delta_{2}\pi$. Substituting (A4) into (A3), $\Phi_{1}$ is written as

(A7) Φ1=1−e−δ0τ4∑i=0m0−1δ0τ4ii!−∑k=0m2−1∑r=0k∑s=0r∑t=0m0−1∑n=0∞krrsm0−1t(−1)n+r+s+m0−t−1k!n!(m0−1)!δ0m0+nδ2kτ3k−r×πreμωs+m0−t−1(−1)N+2φN+1(N+1)!Ei(ψ1)−Ei(ψ2)+∑m=0Neψ1ψ1m(τ4+ω)N+1−eψ2ψ2mωN+1(N+1)N⋯(N+1−m),

where $\mu =\delta_{0}\omega +\delta_{2}\pi -\delta_{2}\tau_{3}$. $\Phi_{2}$ and $\Phi_{3}$ can be calculated as

(A8) $$\begin{array}{c} \Phi_{2}=\mathrm{Pr}\left({\left|{\hat{h}}_{1}\right|}^{2}>\tau_{2}\right)=e^{-\delta_{1}\tau_{2}}\sum_{j=0}^{m_{1}-1}\frac{{\left(\delta_{1}\tau_{2}\right)}^{j}}{j!}, \end{array}$$

(A9) $$\begin{array}{cc} \Phi_{3} & =\mathrm{Pr}\left({\left|{\hat{h}}_{1}\right|}^{2}<\tau_{2},{\left|{\hat{h}}_{0}\right|}^{2}<\tau_{4}\right)\\ & =\mathrm{Pr}\left({\left|{\hat{h}}_{1}\right|}^{2}<\tau_{2}\right)\mathrm{Pr}\left({\left|{\hat{h}}_{0}\right|}^{2}<\tau_{4}\right)\\ & =1-e^{-\delta_{0}\tau_{4}}\sum_{i=0}^{m_{0}-1}\frac{{\left(\delta_{0}\tau_{4}\right)}^{i}}{i!}-e^{-\delta_{1}\tau_{2}}\sum_{j=0}^{m_{1}-1}\frac{{\left(\delta_{1}\tau_{2}\right)}^{j}}{j!}+e^{-\left(\delta_{0}\tau_{4}+\delta_{1}\tau_{2}\right)}\sum_{i=0}^{m_{0}-1}\sum_{j=0}^{m_{1}-1}\frac{{\left(\delta_{0}\tau_{4}\right)}^{i}{\left(\delta_{1}\tau_{2}\right)}^{j}}{i!j!}, \end{array}$$

where $\delta_{1}=\frac{m_{1}d_{1}^{\alpha }}{1-\eta_{1}}$, $\tau_{2}=\frac{\lambda_{1}\gamma_{th_{2}}}{(a_{2}-a_{1}\gamma_{th_{2}})\rho }$, and $\lambda_{1}=\eta_{1}d_{1}^{-\alpha }\rho +1$. Substituting (A7)–(A9) into (A1), (34) can be obtained. The theorem is proved.  □
